# Resource utilization pattern and cost of tuberculosis treatment from the provider and patient perspectives in the state of Penang, Malaysia

**DOI:** 10.1186/1472-6963-14-353

**Published:** 2014-08-19

**Authors:** Muhammad Atif, Syed Azhar Syed Sulaiman, Asrul Akmal Shafie, Muhammad Asif, Zaheer-Ud-Din Babar

**Affiliations:** Department of Pharmacy, The Islamia University of Bahawalpur, Punjab, Pakistan; Discipline of Clinical Pharmacy, School of Pharmaceutical Sciences, Universiti Sains Malaysia, Penang, Malaysia; Discipline of Social and Administrative Pharmacy, School of Pharmaceutical Sciences, Universiti Sains Malaysia, Penang, Malaysia; Department of Pharmacology, School of Pharmaceutical Sciences, Universiti Sains Malaysia, Penang, Malaysia; Division of Pharmacy Practice, School of Pharmacy, University of Auckland, Auckland, New Zealand

**Keywords:** Resource utilization, Cost of tuberculosis treatment, Smear positive pulmonary tuberculosis, Patient cost, Provider cost, Predictors of tuberculosis treatment cost, Malaysia

## Abstract

**Background:**

Studies from both developed and developing countries have demonstrated a considerable fluctuation in the average cost of TB treatment. The objective of this study was to analyze the medical resource utilization among new smear positive pulmonary tuberculosis patients. We also estimated the cost of tuberculosis treatment from the provider and patient perspectives, and identified the significant cost driving factors.

**Methods:**

All new smear positive pulmonary tuberculosis patients who were registered at the chest clinic of the Penang General Hospital, between March 2010 and February 2011, were invited to participate in the study. Provider sector costs were estimated using bottom-up, micro-costing technique. For the calculation of costs from the patients’ perspective, all eligible patients who agreed to participate in the study were interviewed after the intensive phase and subsequently at the end of the treatment by a trained nurse. PASW was used to analyze the data (Predictive Analysis SoftWare, version 19.0, Armonk, NY: IBM Corp.).

**Results:**

During the study period, 226 patients completed the treatment. However, complete costing data were available for 212 patients. The most highly utilized resources were chest X-ray followed by sputum smear examination. Only a smaller proportion of the patients were hospitalized. The average provider sector cost was MYR 992.34 (i.e., USD 325.35 per patient) whereby the average patient sector cost was MYR 1225.80 (i.e., USD 401.90 per patient). The average patient sector cost of our study population accounted for 5.7% of their annual family income. In multiple linear regression analysis, prolonged treatment duration (i.e., > 6 months) was the only predictor of higher provider sector costs whereby higher patient sector costs were determined by greater household income and persistent cough at the end of the intensive phase of the treatment.

**Conclusion:**

In relation to average provider sector cost, our estimates are substantially higher than the budget allocated by the Ministry of Health for the treatment of a tuberculosis case in Malaysia. The expenses borne by the patients and their families on the treatment of the current episode of tuberculosis were not catastrophic for them.

## Background

Prevalence and incidence rates are the most common indicators to weigh the burden of tuberculosis (TB) and highlight the gravity of the epidemic. However, these pointers may fail to explain the trends in the societal and economic burden of the disease [1]. Therefore, it is crucial not only to highlight the importance of an increase in the incidence rates, but also to address the structural and economic barriers which may be acting together to fuel the epidemics [2–4].

The economic impact of TB is often measured in terms of direct and indirect costs to the public health care services. These include the cost of medicines, employees and other health care facilities [5]. Assessing the utilization of health care resources at governmental level is always important to provide long-term planning in a highly dynamic health care system [6]. Furthermore, cost estimates are increasingly required by insurance companies, government payers and others groups which are conscious to their limited research and treatment budget.

In order to fully understand the impacts of TB on the well-being of the members of a society, there is a need to take into account the costs incurred by the patients, their families and communities [7, 8]. Unfortunately, these are occasionally overlooked over the expenses of governmental agencies such as departments of health [5]. As such, many of the government budgets were allocated in a way which did not minimize the burden of the disease [4, 7].

Studies from developing countries have demonstrated that an average cost for the treatment of a drug susceptible TB case ranged from USD 94.00 to USD 2058.00 [4, 8–14]. Similarly, studies from developed countries have shown a considerable fluctuation in the average cost of TB treatment [6, 15]. The high variation of the cost of TB treatment is caused by the different health care systems, perspectives (i.e., provider and patient), cost components, and methods of calculation and data collection used by the respective researchers. This means that this information is neither transferable nor any of the implications arising from these studies can be applied to other health care settings [16].

As a country that is seeing resurgence of TB incidence, understanding of the burden of such disease in the local setting is critical for its successful management. Therefore, the objective of this study was to analyze the medical resource utilization among new smear positive pulmonary tuberculosis (PTB) patients. We also estimated the cost of TB treatment from the provider and patient perspectives, and identified the significant cost driving factors.

## Methods

### Study setting

The study was conducted at the chest clinic of the Penang General Hospital (PGH), which is the first health care facility in Malaysia since 1961. The chest clinic of PGH has eight to nine full-time medical doctors, including three chest consultants. Besides this, the chest clinic has paramedic staff to provide quality care to the patients. The chest clinic has well equipped TB diagnostic laboratory where the specimens of suspected and existing TB patients are investigated using sputum smear examination, culture, nucleic acid amplification tests and drug sensitivity testing (DST). The radiology and pathology departments of the PGH also provide routine investigation services to TB patients.

Confirmed TB patients are advised to take their medication either at the chest clinic or at a primary health care unit. In general, patients are advised to take their daily medicines under the direct observation of a staff nurse at the chest clinic or a primary health care unit. However, some patients are allowed weekly packing of the daily dose. Weekly packing of the daily dose is only available at the chest clinic. The patients who continue their daily treatment at a primary health care unit are advised to visit the chest clinic (every 2 weeks during the intensive phase (IP) and every month during the continuation phase (CP) of the treatment) for routine investigation. Patients who default from their treatment for five consecutive days are traced by a team of staff comprising of a TB-coordinator, a staff nurse and an attendant [17, 18].

### Study design and population

**Figure 1:**
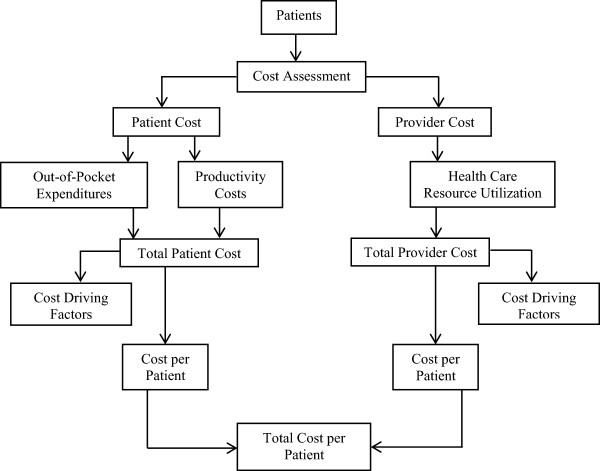
**Outline of the cost analysis.**

A prospective, incidence-based study design was employed [19, 20]. The population in this study consisted of all new smear positive PTB patients who were diagnosed and successfully completed their treatment at the study site between March 2010 and February 2011. The costs attributed to the treatment of TB were estimated from the provider and patient perspectives (Figure 1). Provider costs were estimated using bottom-up, micro-costing technique [21], whereby patient sector costs were estimated by an interviewed-administered questionnaire [4, 22]. The patients who defaulted, transferred-out or died during the treatment were excluded from the study [7, 12].

### Data collection and costing

The medical records of the patients were reviewed for the information on their socio-demographic, clinical and treatment-related characteristics, and resource utilization pattern. To estimate the patient sector costs, a trained nurse invited all eligible patients (i.e., a new case of smear positive PTB) to participate in the study. The nurse explained the purpose of the study and assured the consenting patients about the confidentiality of the information provided. All consented patients were interviewed twice (i.e., after the IP and then subsequently at the end of the treatment/CP) by a trained nurse using an interviewer-administered questionnaire [4, 12, 15, 22]. The respondents who were either unwilling to be interviewed on the second occasion or those who defaulted, died or transferred-out were excluded from the study [12]. Details about the average household monthly income and loss of time due to illness were provided by the patients during the interviews.

#### Provider sector costs

The total provider sector cost was the sum of outpatient clinic costs (i.e., the sum of human resource cost, capital cost, electricity cost and consumables cost), hospitalization costs, chest X-ray costs (i.e., the sum of human resource cost, capital cost, electricity cost and consumables cost [23, 24]), laboratory services costs (i.e., the sum of human resource cost, capital cost, electricity cost and consumables cost) and medicines costs of all patients included in the study. The average provider sector cost was obtained by dividing the total provider cost by the number of successfully treated patients.

To estimate the human resource cost for each service, a time motion study on activity-based-costing (ABC) approach was used. Interviews with the key personnel at the chest clinic and other departments were conducted to identify the principal activities of each service provided to the enrolled patients. This was followed by recording the time taken to complete each activity using a stopwatch. The duration was captured 15 times each for three alternate days and summarized as the mean time (minutes) for each activity. The personnel time cost for each of the employees involved was calculated according to the pay scale of the Federal Civil Services Officers under the System of Remuneration Malaysia [25]. Prior to the calculation, these salaries were converted into the salary per minute (MYR/min) by assuming a daily working time of 8 hours and a monthly working time of 20 days [23, 24]. The cost of each employee per single activity was obtained by multiplying the mean time (minutes) spent by that employee doing a specific activity by his/her salary per minute. Finally, the total human resource cost per service was the sum of human resource cost of all activities involved in that specific service [23, 24].

The costs of the equipment and furniture were obtained from the procurement section of each department. The costs of the building were calculated by multiplying the area size of the service with the unit cost of public building (MYR 85/ft^2^) [24]. The useful life was assumed to be 5 years for clinical equipment and 30 years for the building [26]. Moreover, straight-line deprecation with a discount rate of 3% was used. At the end of the asset’s useful life, the resale value was considered to be 10% of the initial costs [21]. The equivalent annual cost for each asset was calculated based on the following equations:


The unit asset cost was obtained by dividing the equivalent annual cost of each asset by the total number of patients exposed to that service in one year. The total capital cost was the sum of unit costs of all the assets involved in the specific service.

Annual electric power consumption (kW/h) for each of clinical equipment was calculated separately and then multiplied by the unit price of one kW/h (MYR 0.312/kWh) [27]. The resultant annual electricity cost for each of the equipment was then divided by the number of patients exposed to the service (in one year) to obtain the cost per service. The total electricity cost was the sum of unit costs of all equipment involved in the specific service. Likewise, exactly the same method was adopted in estimating the electricity costs of other electrical appliances (e.g., fans, air conditioners, fridge, etc.).

Evidence of consumable expenditure was already available for the majority of the laboratory tests conducted in the various sections of the pathology department. For the remaining tests/services, inclusive information about the utilization of consumables was collected by following the complete activity. In this case, the unit cost of each consumable was obtained from the procurement section of the relevant department. For each test/service, the total cost of consumables was the sum of unit costs of all the consumables used in that specific test/service.

The unit price of the medicines for the total duration of therapy was dependent on the number and quantity of TB drugs and the duration of therapy. The drug costs were obtained from the procurement section of the pharmacy department of the PGH.

The total cost of hospital admission was obtained by multiplying the length of hospital stay (days) by the daily hospitalization cost of MYR 285.00 [28].

#### Patient sector costs

The total patient sector cost was the sum of out-of-pocket expenditures and productivity costs of the patients enrolled in this study. The total patient sector cost was divided by the total number of enrolled patients to arrive at an average patient sector cost.

Out-of-pocket expenditures included prescribed or over-the-counter non-TB medicine (e.g., paracetamol, vitamins, etc.) costs, private medical consultation fees, the amount spent on private laboratory and imaging services (e.g., chest X-rays, liver function tests, etc.), transportation costs (from home to the chest clinic or a primary health care unit and parking/toll charges), special food costs and other costs (i.e., extra telephone bills, hotel stay, etc.). Out-of-pocket expenditures were calculated for the IP and CP of the treatment.

Productivity costs were calculated using the human capital approach as loss of time (days) due to illness. These costs were calculated by using the following equation [22]:


Where;

*20 = number of working days in a month*

The proportion of the average patient sector cost in relation to the annual family income of the patients (affordability assessment) was calculated by using following equation [7, 22]:


Where;

*12 = number of months in a year*

Health expenditures exceeding 10% of family income were considered catastrophic [4, 29].

### Definition of terms

#### Prolonged treatment duration

The total duration of TB treatment longer than 6 months represented prolonged treatment duration [30].

#### High-grade sputum

Sputum was graded according to the number of acid-fast bacilli (AFB) visible at the time of sputum smear microscopy. To simplify the grading categories, four grades (i.e., scanty positive, 1+, 2+ and 3+) were combined to two categories. Scanty positive and 1+ represented low-grade sputum, whereby 2+ and 3+ denoted high-grade sputum [31].

### Statistical analysis

The data was tabulated using Microsoft Excel (2010) and subsequently analyzed by using the PASW (Predictive Analysis SoftWare, version 19.0, Armonk, NY: IBM Corp.). All costs were reported as mean and median. Kolmogorov-Smirnov test was used to test the normality of the distribution of cost data. Costs were found to be non-normally distributed. Simple linear regression analysis (log-transformed costs) was used to examine the possible association between cost and selected socio-demographic and clinical variables. Only the statistically significant variables in the univariate analysis were entered into the multiple linear regression analysis to predict the final independent cost driving factors. The significance of the statistical tests was taken at a p-value of <0.05.

All costs were reported in Malaysian Ringgit (MYR). However, some costs (i.e., the average provider cost, the average patient cost and the total average cost of TB) were also presented in the United States Dollars (USD) for the ease of comparisons with published studies (conversion rate of USD 1 = MYR 3.05).

### Ethical approval

The study was approved by the Medical Research Ethics Committee (MERC), Ministry of Health, Malaysia (MERC reference: dim. KKM/NIHSEC/08/08/04P10-69).

## Results

During the treatment, 226 patients completed their treatment. However, complete costing data were available for 212 patients. The average household income of the study participants was MYR 1789.90 per month. The average duration of TB treatment was 8.18 (SD = 1.64) months (249 days). Tables 1 and 2 provide details about the socio-demographic and clinical characteristics of the patients, respectively.

**Table 1:** **Socio-demographic characteristics of the patients**

Characteristics	Patients n (%)
**Sex**	
Male	142 (67.0)
Female	70 (33.0)
**Age group**	
18-24	18 (8.5)
25-34	21 (9.9)
35-44	39 (18.4)
45-54	49 (23.1)
55-64	49 (23.1)
65-74	26 (12.3)
75+	10 (4.7)
**Ethnicity**	
Malay	64 (30.2)
Chinese	128 (60.4)
Indian	18 (8.5)
Foreigners^*^	2 (0.9)
**Marital status**	
Single	63 (29.7)
Married	125 (59.0)
Widow/divorced	24 (11.3)
**Level of education**	
No education	13 (6.1)
Primary	99 (46.7)
Secondary	70 (33.0)
University	30 (14.2)
**Drug abuser**	
Yes	16 (7.6)
No	196 (92.4)
**Smoker**	
Yes	107 (50.5)
No	105 (49.5)
**Alcoholism**	
Yes	71 (33.5)
No	141 (66.5)
**Employment status**	
Employed	159 (75.0)
Unemployed	53 (25.0)

^*^Indonesian (n = 1), Myanmar (n = 1).

**Table 2:** **Clinical characteristics of the patients**

Characteristics	Patients n (%)
**Form of TB**	
S^+^ PTB^*^	205 (96.7)
S^+^ PTB^*^ and EPTB^**†**^	7 (3.3)
**Sputum grading**	
High-grade	131 (61.8)
Low-grade	81 (38.2)
**Lung cavities**	
Yes	102 (48.1)
No	110 (51.9)
**Persistent cough at the end of the intensive phase**	
Yes	52 (24.5)
No	160 (75.5)
**Persistent cough at the end of the treatment**	
Yes	12 (5.7)
No	200 (94.3)
**Co-morbidities** ^**‡**^	
Diabetes	90 (42.5)
Hypertension	31 (14.6)
Acquired immunodeficiency syndrome	9 (4.2)
Other	28 (13.2)
**Twice-weekly dose**	
Yes	57 (26.9)
No	155 (73.1)
**Prolonged treatment duration (>6 months)**	
Yes	157 (74.1)
No	55 (25.9)

^*^Smear positive pulmonary tuberculosis; ^†^Extrapulmonary tuberculosis; ^‡^Percentages calculated on the basis of N = 212.

### Provider sector costs

Table 3 describes a complete picture of TB-related medical resource utilization pattern.

**Table 3:** **Resource utilization pattern**

Resource	Total utilizations (% of patients)	Mean utilizations (median)
**Chest Clinic**		
Scheduled consultation visits	1683 (100)	7.94 (8)
Visits for medicine (DOT^*^ room visits)	30931 (100)	145.9 (160)
Consultations with the specialist (head of the department)	62 (27.4)	0.99 (1)
Consultations with the specialist	116 (45.3)	1.21 (1)
Consultations with the medical officer	1561 (100)	3.56 (3)
TB coordinator	848 (100)	4 (4)
Counter clerk and attendant	212 (100)	1 (1)
Lab. investigation attendant	1107 (100)	2.65 (3)
**Hospitalization**	77 (11.8)	0.36 (0)
**Chest X-ray**	1233 (100)	5.82 (6)
**Laboratory tests**		
**Bacteriological tests**		
Sputum smear	667 (100)	3.15 (3)
Culture	165 (71.7)	0.78 (1)
Polymerase chain reaction	152 (69.3)	0.72 (1)
Drug sensitivity testing	150 (68.4)	0.71 (1)
Non-specific tests		
Biochemical	839 (99.5)	3.96 (3)
Hematological	421 (93.9)	1.99 (2)
Serological	279 (86.8)	1.32 (1)

^*^DOT: Directly Observed Treatment.

The total provider sector cost for the successful treatment of 212 new smear positive PTB patients was MYR 210376.93. Outpatient clinic costs constituted the highest proportion (38.7%) of the total cost followed by the medicine costs (21.6%). The average provider sector cost was MYR 992.34 (median = MYR 891.55) (USD 325.35 per patient). Table 4 provides details about the provider sector cost.

**Table 4:** **Total provider sector cost**

Cost element	Total cost (intensive phase)	Total cost (continuation phase)	Total cost (% of total cost)	Cost per patient
**Outpatient clinic**				384.44
***Human resource***			57004.58 (69.9)	
Scheduled consultation visits	7668.14	6149.29	13817.43	
Visits for medication (DOT^*^ room visits)	8331.40	15485.47	23816.87	
Consultations with the specialist	1008.60	33.62	1042.22	
Consultations with the specialist (head of the department)	887.40	-	887.40	
Consultations with the medical officer	6795.73	6670.71	13466.44	
TB coordinator	1539.12	1539.12	3078.24	
Counter clerk and attendant	165.36	-	165.36	
Lab. investigation attendant	508.20	222.42	730.62	
***Capital***	3137.80	5832.09	8969.89 (11.0)	
***Electricity***	5085.40	9452.01	14537.41 (17.8)	
***Consumables***	946.28	43.53	989.81 (1.2)	
**Sub-total**			**81501.69 (38.7)**	
**Hospitalization**	22022.00	-	**22022.00 (10.5)**	103.88
**Chest X-ray**	4480.45	2486.00	**6966.45 (3.3)**	32.86
**Bacteriological tests**				
Sputum smear	2155.71	492.28	2647.99 (6.7)	
Culture	1323.30	-	1323.30 (3.4)	
Polymerase chain reaction	15493.36	-	15493.36 (39.5)	
Drug sensitivity testing	19791.00	-	19791.00 (50.4)	
**Sub-total**			**39255.65 (18.7)**	185.17
**Non-specific laboratory tests**	12946.43	2139.70	**15086.13 (7.2)**	71.16
**Medicines**	25069.95	20475.06	**45545.01 (21.6)**	214.83
**Total Cost**	**139355.63**	**71021.30**	**210376.93 (100)**	**992.34**

^*^DOT: Directly Observed Treatment.

Note: All costs in Malaysian Ringgit. Bold numbers reflect sub-total of cost components, grand total cost and cost per patient.

### Patient sector costs

Out of 212 eligible patients, 198 agreed to participate in the study. Among the consented patients, six (9.4%) dropped-out from the second interview. Therefore, the usable sample size for patient sector costs was 192 patients. Most of the out-of-pocket expenditures were made on transportation (40%) and special food (29.5%). The total out-of-pocket expenditures per patient were MYR 439.42 (median = MYR 345.00) (Table 5).

**Table 5:** **Total out-of-pocket expenditures**

Cost element	Cost during intensive phase	Cost during continuation phase	Total cost (% of total cost)	Cost per patient
Consultation fees	4184.00	2771.00	6955.00 (8.2)	36.22
Chest X-ray	1324.00	785.00	2109.00 (2.5)	10.98
Non-TB medicines	3662.00	85.00	3747.00 (4.4)	19.52
Laboratory tests	1956.00	2331.00	4287.00 (5.1)	22.33
Traditional medicine	3653.00	2614.00	6267.00 (7.4)	32.64
Transportation	15790.00	17939.00	33729.00 (40.0)	175.67
Special food	15533.00	9319.00	24852.00 (29.5)	129.44
Other	480.00	1942.00	2422.00 (2.9)	12.61
**Total out-of-pocket expenditures**			**84368.00 (100)**	**439.42**

Note: All costs in Malaysian Ringgit.

On an average, each patient lost 10 days (median = 8 days; range = 3–29 days) of normal productivity during the complete course of treatment. The total productivity cost for all patients (N = 192) was MYR 150,985.40, while the average productivity cost was MYR 786.38 (median = MYR 515.75).

The total patient sector cost (N = 192), derived as the sum of out-of-pocket expenditures and productivity costs, was MYR 235,353.40 (Table 6). The average patient sector cost was MYR 1,225.80 (median = MYR 951.13) (USD 401.90 per patient). The average patient sector cost of our study population accounted for 5.7% of their annual family income.

**Table 6:** **Total patient sector cost**

Cost element	Total patient cost (%)	Cost per patient
Out-of-pocket expenditures	84368 (35.9)	439.42
Productivity costs	150985.40 (64.1)	786.38
**Total cost**	**235353.40 (100)**	**1225.80**

Note: All costs in Malaysian Ringgit.

### Total average cost of tuberculosis treatment

The total average cost of TB treatment (provider and patient perspective) was MYR 2,218.14 (USD 727.24 per patient). Table 7 shows that the amount paid by the patient constituted 55.3% of the total average cost.

**Table 7:** **Cost of tuberculosis treatment per patient treated**

Perspective	Cost per patient	% from total cost
Provider	992.34	44.7
Patient	1225.80	55.3
**Total cost**	**2218.14**	**100**

Note: All costs in Malaysian Ringgit.

### Provider and patient sector cost driving factors

Table 8 describes the provider and patient sector cost driving factors using a multiple linear regression analysis. Provider costs were higher among the patients with treatment duration more than 6 months (p = 0.002). However, lower provider costs were associated with twice-weekly dosing schedule (p <.0005). Similarly, higher patient costs were associated with greater household monthly income (p <.0005) and persistent cough at the end of the IP of the treatment (p = 0.004). However, patient costs were significantly lower among the unemployed patients (p = 0.003).

**Table 8:** **Final provider and patient sector cost driving factors: multiple linear regression analysis with log-transformed costs**

Independent variables	***B***	***S.E***	***p***-value
Provider sector*			
Indian ethnicity	.114	0.032	.071
High-grade sputum	.122	0.021	.057
Lung cavities	.099	0.020	.126
Twice-weekly dose	-.267	0.022	**<.0005**
Prolonged treatment duration	.207	0.022	**.002**
**Patient sector** ^**†**^			
Age^‡^	-.084	0.002	.219
Widow/divorced	-.001	0.070	.994
University education	.029	0.074	.685
Unemployment	-.195	0.055	**.003**
Household income^‡^	.515	0.001	**<.0005**
Persistent cough at the end of intensive phase	.178	0.048	**.004**

^*^Model summary: R^2^ = .286, *p* <.0005; ^**†**^Model summary: R^2^ = .419, *p* <.0005; ^‡^Denotes continuous variable.

Note: Only statistically significant variables in univariate analysis were entered in multiple linear regression analysis and are shown in the table.

## Discussion

Assessing the utilization of health care resources is critical for long-term planning in the changing health care system and evaluating cost-effective strategies [6]. Our findings showed 145.9 directly observed treatment (DOT) visits per patient. In contrast, a Haitian study reported 75 DOT visits per patient [32]. Along the same lines, studies from Tanzania and Tajikistan mentioned fewer than 80 DOT visits per patient [11, 12]. Nonetheless, these differences in DOT visits might be associated with difference in treatment strategies (i.e., daily dose vs. twice-weekly dose, DOT at clinic vs. weekly packing of the daily dose) employed at these settings. Alternatively, variations in the average treatment duration might be another reason for such differences.

In this study, the proportion of hospitalized patients was quite low (11.8%). Likewise, a study from Zambia reported only 6% hospitalization rate among TB suspects whereas no patient was hospitalized after confirmed diagnosis of TB [10]. On the other hand, studies from the Netherlands, Haiti, Tajikistan and India reported 25–75% hospitalization rates among TB patients [12, 22, 32, 33]. In our study, lower number of hospitalizations was a consequence of the local health care policy of not admitting new smear positive PTB patients in the hospital wards. Appropriate treatment of smear positive index cases could render most of them non-infectious, usually within 2 weeks. Additionally, the infectivity could be significantly decreased within 2 days of standard TB therapy. Hence, hospitalization should only be reserved for severely ill TB patients (e.g., severe cough and high fevers) and for those who are unable to take care of themselves.

In our study, all enrolled patients were investigated and monitored through chest roentgenogram. The average number of chest X-ray films per patient was 5.82. Studies from Thailand and Lusaka reported an average of two chest X-ray films per patient [4, 34]. In our study, most of the patients did not produce sputum for bacteriology after 1–2 months of TB treatment. As a result, the clinicians were unable to monitor the treatment progress through smear microscopy. Consequently, they had to monitor the treatment progress through frequent chest X-rays. However, according to World Health Organization (WHO), it is pointless, unreliable and wasteful of resources to monitor the patient through the use of chest X-rays [30].

Cost and cost-effectiveness studies from Tanzania, Uganda and South Africa emphasized the importance of implementing community-based TB care [9–11]. A systematic review which compared self-administered therapy with DOT concluded that DOT did not improve the outcomes [35]. On the contrary, other review articles suggested DOT to be associated with higher treatment success rates [36, 37]. In line with the findings of above-cited studies and owing to higher outpatient clinic costs in our study (i.e., 38.7% of the total provider sector cost), community involvement appears to be an attractive economic option. A network of strong community-based TB care may be attained through active involvement of NGOs (non-governmental organizations) and treatment supporters. Cured TB patients can be the best treatment supporters, as can friends, family members, neighbors, religious leaders and holistic practitioners [30, 38]. We also believe that DOT room visits and associated costs could be reduced by adopting WHO recommended three times a week therapy during the CP of the treatment. Alternatively, weekly packing of the daily dose could be an option to reduce the DOT room visit costs.

In this study, the cost of bacteriological tests constituted 18.7% of the total provider cost. A notable proportion (89.9%) of this cost was shared by DST and polymerase chain reaction (PCR). As per the recommendations of WHO, DST should be performed in settings where country data (or WHO estimates) suggests multidrug-resistant tuberculosis (MDR-TB) in more than 3% of new patients. WHO also recommends performing culture and DST in new TB patients (from areas with <3% MDR-TB rates) if they are still positive for AFB on direct smears at the end of the third month of TB treatment. According to a recent TB country profile, the percentage of TB cases with MDR-TB was only 0.1% in Malaysia [39]. Using these facts and recommendations by WHO [30], a strategy of limiting the use of DST and PCR for the re-treatment cases or for those who are still smear positive at the end of the third month of treatment could significantly reduce the provider cost in the local settings (Note: unit cost of DST = MYR 131.94, unit cost of PCR = MYR 101.93). Earlier studies also reported that most of the lower-middle income countries did not prefer these tests for new smear positive PTB patients owing to higher costs [10, 33, 34].

The cost of TB medicines was the second highest cost component after the outpatient clinic costs in that it constituted 21.6% of the total provider sector cost. These results are somewhat comparable to the findings of similar studies published elsewhere [10, 13]. However, a few studies from developing and developed countries showed that the cost of TB medicines ranged from 4.7–63.8% of the total cost of TB management [6, 9–11, 14, 32]. Differences in cost accounting methods, characteristics of study population and variability in the unit cost of TB medicines are some of the factors that could explain inconsistency in the findings of various studies.

With regard to patient sector costs, out-of-pocket expenditures constituted 35.9% of the total cost to the patients. Among these out-of-pocket payments, transportation and special food costs created the highest impact. Consistent with our findings, studies from the Netherlands, Tajikistan, Yemen, India and Thailand reported higher transportation and special food costs [7, 12, 14, 22, 33]. As such, weekly packing of TB drugs in the presence of suitable treatment supporters could be an option to reduce the out-of-pocket costs to the patients.

In this study, on an average, each patient lost 10 days of normal productivity. Conversely, TB patients lost an average of 135 working days in Tajikistan [12]. Similarly, average work time loss in TB patients was 81 days in the Netherlands [22]. Differences in the work time loss between these studies might be attributed to the policy of compulsory hospitalization of smear positive PTB patients in some countries. In fact, a greater proportion of the patients were hospitalized in above-cited studies from Tajikistan and the Netherlands.

The average patient sector cost of our study population accounted for 5.7% of their annual family income. This is an indication that these expenses were not catastrophic for them. However, these costs could be a considerable portion of their disposable income after payment of all monthly fixed costs such as rentals, monthly installments, bills (i.e., electricity, water and gas) and insurances. A study from Thailand reported that expenses borne by TB patients ranged from 5.1–20.3% based on different income groups [7]. However, existing literature reported that the costs borne by TB patients could be as high as 75% of their annual household income [22]. The differences among the findings of various studies might be the consequence of differences in the socioeconomic status of study participants. Another possibility could be differences in the accessibility of health care services irrespective of ability to pay. In Malaysia, TB patients are neither charged for diagnostic services, nor for TB medicines. Furthermore, TB patients are given top priority in terms of consultations with the medical practitioners. In addition, TB patients are further facilitated by providing routine therapy at the primary health care units. All these measures are taken to substantially reduce the patient sector costs to an extent that these are not catastrophic for them and/or their families. The results of this study add weight to the appropriateness of all measures taken to alleviate the financial burden of the disease on patients.

According to our estimates, the total average cost of treatment for a new smear positive PTB patient was MYR 2,218.14 (USD 727.26). The average patient cost was slightly higher (54.3%) than the provider cost. Recently, a study from Western Asia showed that the average cost of treating a PTB patient was USD 142.40, in which patient costs constituted 76.1% of the total cost [14]. In 2005, a Tanzania study reported that on an average USD 145.00 were required to treat an episode of TB, whereby the provider costs constituted 70.3% of the total cost [11]. Another study from Africa showed that an average of USD 703.00 was required to treat a PTB patient [10]. In summary, the total average cost of TB treatment varied noticeably among various studies as did the distribution between the health services and the patients.

In multiple linear regression analysis, higher provider costs were associated with longer treatment duration. This is an indication that the patients in whom the treatment was prolonged had utilized the health care resources for a relatively longer period of time. In contrast, twice-weekly dosing schedule during the CP of the treatment was the only factor inversely related to higher provider costs. A possible explanation for this association could be relatively lower utilization of DOT room for the collection of TB medicines.

Highly educated individuals are socially advantaged, and thus are more likely to seek frequent medical care at private health facilities. In turn, they have to bear more out-of-pocket expenditures [40]. Similarly, patients with persistent signs and symptoms are more likely to spend on their health in the form of consultations with private medical practitioners and/or use of complementary and alternative medicines. In accordance with these arguments, we also found a similar relationship between patient-related costs and our study variables.

Our study has three limitations. First, in this study, we only included smear positive PTB patients. The cost of retreatment cases and those with MDR-TB is sometimes substantial [6]. Therefore, our results could not be generalized for all forms of TB patients. However, from the public health perspective, smear positive PTB patients are the most important group of TB patients, and these are the most common group of patients used to evaluate the performance of National Tuberculosis Program. Second, costs reported by the patients could have been biased because of patients’ failure to recall certain out-of-pocket expenditures and productivity losses. We assume that this recall bias could lead to underestimation or to some extent overestimation of patient sector costs. However, we helped the patients in their recall work using a calendar of locally important events. In the event of reporting unexpectedly high or low costs, patients were asked again to confirm the accuracy of information. We strongly believe that these measures could have significantly reduced the recall bias, and our cost estimates are reliable. Finally, we conducted our study in a public sector hospital where TB patients are treated free of cost. Therefore, cost structure could be substantially different for the patients who seek care in the private health care sector where they are charged based on the type and frequency of service utilized.

## Conclusion

Our findings could be used to predict medical utilization for the diagnosis and treatment of TB in patients seeking care in a public sector hospital. In relation to average provider sector cost, our estimates are substantially higher than the budget allocated by the Ministry of Health for the treatment of a TB case in Malaysia (i.e., USD 325.35 vs. USD 225.00). We also found that TB diagnosis and treatment did not pose a significant economic burden on the patients and their families. Community-based DOT, limited use of DST and PCR, and special attention to the cost driving factors are some of the options to reduce cost of TB treatment. However, future studies are required to warrant these statements.
